# To the editor: Response to post-infection cognitive impairments in a cohort of elderly patients with COVID-19, by Wang, Y.J. et al. (2021)

**DOI:** 10.1186/s13024-022-00567-3

**Published:** 2022-09-25

**Authors:** Rahmouni Nesrine, Rosa-Neto Pedro, Brunet Alain

**Affiliations:** 1 Translational Neuroimaging Laboratory, McGill Research Centre for Studies in Aging, Montreal, Canada; 2 Department of Psychiatry, Faculty of Medicine, Douglas Hospital, McGill University, 6825 La Salle Blvd, Montreal, QC H4H 1R3 Canada; 3 Department of Neurology and Neurosurgery, Faculty of Medicine, McGill University, Montreal, Canada; 4 Douglas Mental Health University Institute, Montreal, Canada

To the Editor,

We have read with great interest the article *Post-infection cognitive impairments in a cohort of elderly patients with COVID-19* by Wang, et al. [1] aiming to evaluate the long-term impact of COVID-19 on patients’ cognition and cognitive decline. The findings suggest that COVID-19 infection is associated with a higher risk of cognitive decline in elderlies in comparison to an uninfected control group. The authors provided an exhaustive list of risk factors of cognitive decline measured in a sample of COVID-19 patients and controls. Given the importance of this public health issue, we wish to raise some methodological considerations limiting the interpretation of certain results, particularly the choice of the control group, the selection of the patients’ informant and the impact of the patients’ lifestyle on the findings.

Using spouses as a control group sounds like a good idea at first sight. However, the fact that spouses had to be in better health conditions for them not to catch COVID-19 when their partner did constitute an important bias. Over a third of the COVID-19 patients in this study had their spouses in the control group. The authors acknowledged this issue as one of their limitations and suggested that non-covid pneumonia patients would have been a better option to assess the COVID-19 specificity of the associations. We agree in principle but think that this wouldn’t be sufficient: Cheng et al. [2] reported a longer Intensive Care Unit (ICU) or hospital stay contributes significantly to the development of dementia in patients. Additionally, Katon et al. [3] reported similar results, showing that older adults who experienced acute and critical illness hospitalization had greater chances of cognitive decline compared with patients that were not hospitalized. In the present paper, the authors report that ICU admission is significantly associated with cognitive impairment and cognitive decline, and severe COVID-19 cases had higher frequency of ICU admission than non-severe COVID-19 cases and controls. For the authors to consider that the cognitive impairment and decline of patients in this study are due to their COVID-19 infection is far-fetched as it can be explained by their ICU admission [4]. A more adequate control group for this study would have been a non-COVID-19 ICU patient.

Furthermore, the characterization of the informant who assessed the patient’s cognitive decline using the Chinese version of the short form Informant Questionnaire on Cognitive Decline in the Elderly (IQCODE) remained elusive. The authors don’t report how this person was chosen, if the patient and their spouse had the same informant, if they were close and had regular contact to be able to report a reliable opinion on the cognitive decline of the patient over the 6-month period. Moreover, in the affected sample, 238 patients had severe covid and 1301 had non-severe covid. Among the informants, one wonders how many had a partner with severe or non-severe covid, as this information is not disclosed in the paper. In addition, the use of the IQCODE as a measure of cognitive decline makes this study a cross-sectional (retrospective) one, not a longitudinal study as it is falsely presented. As the recruitment of the participant was done upon discharge, the TICS-40 could have been administered upon discharge and again after 6 months in order to evaluate the cognitive decline objectively and longitudinally.

Although the paper focuses on cognitive changes, we are wondering about daily functioning: an important factor unassessed in this study is the effect of sleep on cognition. As Hoang et al. [5] suggested, sleep-wake cycle is strongly correlated with brain aging. In older adults, sleep disruption is associated with poorer performance across multiple cognitive domains and more risk of cognitive decline. The reason why the consideration of sleep is so important resides in the fact that, as shown by Vitiello et al. [6], the COVID-19 pandemic has had a high prevalence of sleep problems, which affected about 40% of the general population. This appears to be especially true for those with active COVID-19. This was not examined, unfortunately.

In the end, although this study claims to provide important insights regarding the impact of COVID-19 in cognition, our enthusiasm is tempered by these methodological shortcomings limiting the claims made in this manuscript.

## Data Availability

Not applicable.

## References

[1] Liu YH, Wang YR, Wang QH, Chen Y, Chen X, Li Y, et al. Post-infection cognitive impairments in a cohort of elderly patients with COVID-19. Mol Neurodegener. 2021. 10.1186/s13024-021-00469-w PMID: 34281568; PMCID: PMC8287105. 10.1186/s13024-021-00469-w PMC8287105 34281568

[2] Lai CC, Ho CH, Chen CM, Chiang SR, Chao CM, Liu WL, Lin YC, Wang JJ, Cheng KC (2017). Long-term risk of dementia after acute respiratory failure requiring intensive care unit admission. PLoS One.

[3] Davydow DS, Zatzick D, Hough CL, Katon WJ. In-hospital acute stress symptoms are associated with impairment in cognition 1 year after intensive care unit admission. Ann Am Thorac Soc. 2013;(5):450–7. 10.1513/AnnalsATS.201303-060OC PMID: 23987665; PMCID: PMC3960914. 10.1513/AnnalsATS.201303-060OC PMC3960914 23987665

[4] Ehlenbach WJ, Hough CL, Crane PK, Haneuse SJ, Carson SS, Curtis JR, Larson EB (2010). Association between acute care and critical illness hospitalization and cognitive function in older adults. JAMA..

[5] Yaffe K, Falvey CM, Hoang T (2014). Connections between sleep and cognition in older adults. Lancet Neurol.

[6] Jahrami H, BaHammam AS, Bragazzi NL, Saif Z, Faris M, Vitiello MV (2021). Sleep problems during the COVID-19 pandemic by population: a systematic review and meta-analysis. J Clin Sleep Med.

